# High-Altitude Hypoxia Injury: Systemic Mechanisms and Intervention Strategies on Immune and Inflammatory Responses

**DOI:** 10.3390/antiox15010036

**Published:** 2025-12-26

**Authors:** Jingman Zhang, Shujie Guo, Beiebei Dou, Yang Liu, Xiaonan Wang, Yingze Jiao, Qianwen Li, Yan Li, Han Chen

**Affiliations:** 1 Food Laboratory of Zhongyuan, Henan University of Technology, Zhengzhou 450001, China; jm_zhang@stu.haut.edu.cn (J.Z.); guoshujie@stu.haut.edu.cn (S.G.); 2 Food Laboratory of Zhongyuan, Luohe 462000, China; doubeibei@zyfoodlab.cn (B.D.); liuyang@zyfoodlab.cn (Y.L.); wangxiaonan@zyfoodlab.cn (X.W.); jiaoyingze@zyfoodlab.cn (Y.J.); liqianwen@zyfoodlab.cn (Q.L.); liyan@zyfoodlab.cn (Y.L.); 3 Food Laboratory of Zhongyuan, China Agricultural University, Beijing 100080, China

**Keywords:** high-altitude hypoxia injury, oxidative stress, inflammatory response, co-related injury mechanisms, intervention strategy

## Abstract

High-altitude exposure poses significant health challenges to mountaineers, military personnel, travelers, and indigenous residents. Altitude-related illnesses encompass acute conditions such as acute mountain sickness (AMS), high-altitude pulmonary edema (HAPE), and high-altitude cerebral edema (HACE), and chronic manifestations like chronic mountain sickness (CMS). Hypobaric hypoxia induces oxidative stress and inflammatory cascades, causing alterations in multiple organ systems through co-related amplification mechanisms. Therefore, this review aims to systematically discuss the injury mechanisms and comprehensive intervention strategies involved in high-altitude diseases. In summary, these pathologies involve key damage pathways: oxidative stress activates inflammatory pathways through NF-κB and NOD-like receptor thermal protein domain-associated protein 3 (NLRP3) inflammasomes; energy depletion impairs calcium homeostasis, leading to cellular calcium overload; mitochondrial dysfunction amplifies injury through mitochondrial permeability transition pore (mPTP) opening and apoptotic factor release. These mechanisms could be converged in organ-specific patterns—blood–brain barrier disruption in HACE, stress failure in HAPE, and right heart dysfunction in chronic exposure. Promising strategies include multi-level therapeutic approaches targeting oxygenation (supplemental oxygen, acetazolamide), specific pathway modulation (antioxidants, calcium channel blockers, HIF-1α regulators), and damage repair (glucocorticoids). Notably, functional foods show significant therapeutic potential: dietary nitrates (beetroot) enhance oxygen delivery, tea polyphenols and anthocyanins (black goji berry) provide antioxidant effects, and traditional herbal bioactives (astragaloside, ginsenosides) offer multi-targeted organ protection.

## 1. Introduction

High-altitude areas, marked by low oxygen levels, present notable challenges for a growing number of people, such as mountaineers, travelers [1], military units [2], and local residents. As altitude increases, the partial pressure of oxygen decreases, which can significantly impact the body’s physiological functions and physical performance, leading to various health problems in multiple organs and systems. Acute mountain sickness (AMS) usually occurs in people who ascend rapidly to high-altitude areas without adequate acclimatization, and symptoms may be exacerbated by pre-existing cardiopulmonary disease [3,4]. In severe cases, this can escalate to life-threatening conditions like high-altitude pulmonary edema (HAPE) [5] or cerebral edema (HACE). Clinically, these conditions exhibit distinct altitude thresholds. AMS is generally the first to appear in unacclimatized individuals ascending above 2500 m. HAPE typically manifests at altitudes exceeding 3000 m and is the most common cause of death related to high altitude. In contrast, HACE usually develops at higher elevations, typically above 3500 m, or as a severe progression of AMS. Although overlap exists, HAPE tends to occur at lower altitudes compared to the isolated presentation of HACE [1]. On the other hand, chronic mountain sickness (CMS) predominantly affects populations permanently residing in high-altitude areas rather than those with transient exposure [6], which involves excessive red blood cell production and increased pulmonary arterial pressure [7], considerably affecting a person’s quality of life and overall health.

A wide range of studies have investigated the mechanisms of high-altitude hypoxia injury from different perspectives, including oxidative stress, energy metabolism, vascular adaptation, and mitochondrial function [8,9,10,11]. However, reviews specifically focusing on the immune and inflammatory responses underlying high-altitude hypoxia injury remain scarce. Therefore, the present review seeks to systematically summarize how immune and inflammatory signaling, particularly via ROS, NF-κB, and NLRP3 activation, interacts with calcium dysregulation and mitochondrial dysfunction to exacerbate multi-organ damage. In addition, we discuss how these mechanisms interface with broader adaptive pathways, including nitric oxide signaling and erythropoietin (EPO) regulation, among others. Finally, we integrate these interrelated mechanisms with current therapeutic and nutritional strategies, providing a multi-level framework for understanding and mitigating high-altitude hypoxia injury.

## 2. Hypoxia Definition and Classification

The nature of hypoxia is that tissues do not receive enough oxygen to maintain their regular cellular activity and function. Hypoxia and hypoxemia are not the same concept; hypoxemia is a decrease in the partial pressure of arterial oxygen (PaO_2_), which is a common trigger of hypoxia but not the only factor [12,13]. In some cases, tissues may experience hypoxia even when PaO_2_ appears normal, especially if the blood’s ability to carry oxygen is compromised or if the cells’ capacity to use oxygen effectively is impaired.

From a pathophysiological perspective, hypoxia is typically categorized into four main types [14,15]. These classifications are based on potential issues in the processes of oxygen transport and utilization, as detailed in Table 1.

The high-altitude environment triggers hypoxic hypoxia, with a range of physiological responses and sequelae. This initial injury triggers a cascade of responses such as altered blood viscosity, the development of hemodynamic compensatory erythrocytosis, and, consequently, microcirculatory dysfunction. Hypoxic hypoxia, together with other types of hypoxia-induced conditions, creates a complex pathological basis for a co-related mechanism of injury. In addition, the duration of high-altitude exposure, whether acute or chronic, determines different physiological and pathologic outcomes, as shown in Table 2.

This pathophysiological distinction is fundamental to understanding the progression of high-altitude-related illnesses. In contrast, chronic hypoxia is persistent, with the body’s efforts to adapt continually offset by the slow progression of deleterious physiological changes, both of which are driven by the long-term dysregulation of the same molecular pathways. Thus, a direct decrease in oxygen partial pressure leads to an unusually complex and interrelated systemic injury, and this multilevel pathophysiology provides a crucial basis for studying the intricate co-related mechanisms.

## 3. Co-Related Mechanisms of Hypoxia-Induced Injury in the Body

High-altitude hypoxia triggers a complex cascade of reactions involving a range of molecular and cellular processes that exacerbate tissue damage in various organs. In this section, we will explore the main mechanisms at play, such as oxidative stress, inflammation, calcium imbalance, and mitochondrial dysfunction, and describe how these factors interact to lead to the development of high-altitude-related diseases.

### 3.1. Oxidative Stress and ROS Signaling

High-altitude hypoxia presents a profound physiological paradox: a state of oxygen deficiency that paradoxically triggers a surge in reactive oxygen species (ROS), leading to significant oxidative stress. This phenomenon is commonly referred to as “hypoxic oxidative stress” and is a key initiating event in the pathological cascade of high-altitude disease. The current best definition of oxidative stress is “a transient or long-term increase in steady-state ROS levels, disturbing cellular metabolic and signaling pathways, particularly ROS-based ones, and leading to oxidative modifications of an organism’s macromolecules that, if not counterbalanced, may culminate in cell death via necrosis or apoptosis [32]”.

Under hypoxic conditions, the major sources of ROS are multifaceted. First, oxygen supply is reduced, and the mitochondrial electron transport chain (ETC) becomes an important source. Hypoxia leads to electron accumulation in upstream complexes (especially complexes I and III) through inhibition of cytochrome c oxidase (complex IV), leaving oxygen incompletely reduced, resulting in the production of superoxide anion (O_2_^−^) [33]. Secondly, activation of the enzyme system plays an important role. NADPH oxidase (NOX), which is present in various cells, including endothelial cells and phagocytes, can be directly stimulated by hypoxic signals [34,35,36]. These enzymes are the main source of ROS, independent of the state of the mitochondria. In addition, xanthine oxidase (XO)–mediated ROS generation may occur under certain hypoxic contexts, where conversion of xanthine dehydrogenase to XO is enhanced, and then produces hydrogen peroxide (H_2_O_2_) and superoxide anion (O_2_^−^) during purine catabolism [37]. However, its contribution to sustained hypobaric hypoxia remains debated. For example, Sen and Benoit [38], using the Amplex Ultrared/Horseradish Peroxidase Assay, found that the release of H_2_O_2_ was decreasing, rather than increasing, in HEK293 cells under acute hypoxia, at times ranging from 1 min to 3 h. This contradicts previous conclusions and requires further study.

At the same time, the body’s antioxidant defense system is impaired, and the oxidative load is further increased. Under sustained hypoxic conditions, the activity and expression of key antioxidant enzymes such as superoxide dismutase (SOD), catalase (CAT) and glutathione peroxidase (GPx) are often inhibited, impairing the body’s ability to scavenge ROS [39,40]. Excessive ROS can directly damage cellular components, leading to lipid peroxidation of membranes, protein denaturation and DNA strand breaks [41,42]. However, in addition to this, ROS are also signaling molecules, triggering a variety of pathological effects, acting as second messengers [43,44], activating inflammatory pathways and disrupting intracellular ionic homeostasis [45,46], effectively linking oxidative stress to hypoxic damage. Thus, oxidative stress is not just isolated but is a key factor that amplifies hypoxia-induced damage. It activates complex signaling pathways, especially those associated with inflammatory processes, which we will discuss in more depth in subsequent chapters.

### 3.2. NF-κB and NLRP3-Mediated Inflammation

The inflammatory response does not just occur in conjunction with oxidative stress; it is fundamentally driven and exacerbated by hypoxia itself, which initiates a self-reinforcing cycle of damage through precise oxygen-sensing mechanisms. The central regulatory hub for the inflammatory response under hypoxic conditions is the nuclear factor kappa-light-chain-enhancer of activated B cells (NF-κB). According to the literature, hypoxia activates NF-κB through at least two interconnected pathways.

**Figure 1:**
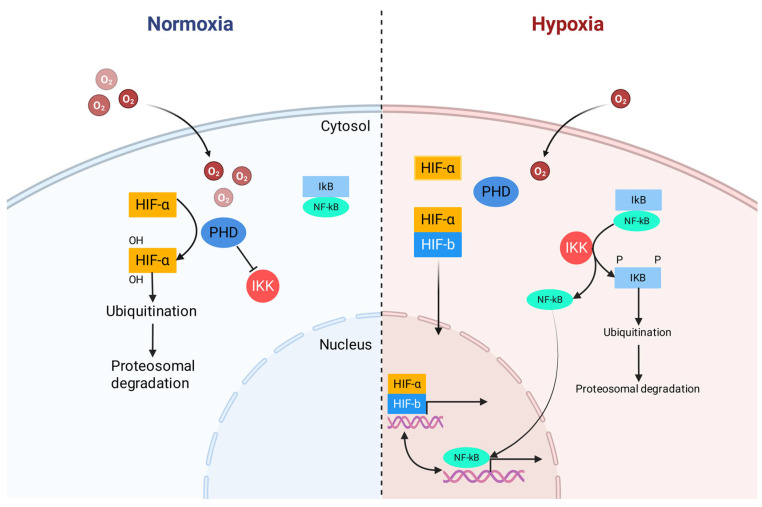
Crosstalk between hypoxia-inducible factor (HIF) and nuclear factor-kappa B (NF-κB). Redrawn and adapted from [48], under CC BY 4.0 license.

First, hypoxia inactivates prolyl hydroxylases (PHDs). These enzymes not only regulate hypoxia-inducible factor (HIF) but also directly control the IκB kinase (IKK) complex upstream of NF-κB. Decreased PHD-catalyzed prolyl hydroxylation relieves the suppression of IKKβ, leading it to phosphorylate IκB, causing its ubiquitination and proteasomal degradation, which ultimately liberates NF-κB to translocate to the nucleus, as shown in Figure 1 [47,48]. Additionally, reactive oxygen species (ROS) produced during hypoxic stress act as important signaling molecules that can also activate the IKK complex, triggering the same downstream events [45,49]. Once in the nucleus, NF-κB promotes the production of several pro-inflammatory cytokines, including tumor necrosis factor-α (TNF-α), interleukin-1β (IL-1β), and interleukin-6 (IL-6). Critically, a powerful bidirectional positive feedback loop is established between hypoxia and inflammation. Activated NF-κB can, in turn, regulate the transcription of HIF-1α, thereby initiating the hypoxic response program even before tissues become severely hypoxic [47,50].

In addition, ROS and their resulting molecular damage products are themselves key damage-associated molecular patterns (DAMPs) [51]. These DAMPs, along with mitochondrial DNA (mtDNA) released as a result of mitochondrial dysfunction, can be recognized by pattern recognition receptors (TLR4) on the surface of immune cells. This is a key trigger for the assembly and activation of NLRP3 inflammasome, a multiprotein signaling hub [52]. Activation of NLRP3 inflammasome cleaves pro-caspase-1 to active caspase-1, which further processes pro-IL-1β and pro-IL-18 into mature, highly bioactive cytokines [53]. Ultimately, oxidative stress is again directly coupled to the inflammatory cascade at the level of NLRP3, forming a second pro-inflammatory hub pathway [54]. More importantly, this interaction is not just unidirectional; inflammatory cytokines produced via ROS-dependent pathways, in turn, exacerbate oxidative stress. For example, TNF-α can promote ROS generation by activating NOXs in different cell types [55]. Similarly, neutrophils that are recruited and activated as a marker of inflammation amplify oxidative damage by generating an oxidative burst through their own NOX enzyme system, releasing large amounts of ROS into the local microenvironment [56,57]. Crucially, these mechanistic pathways identified in animal models have been validated in human clinical studies. Research has demonstrated that exposure to hypobaric hypoxia significantly elevates plasma levels of key pro-inflammatory cytokines, including TNF-α, IL-1β, and IL-6 in human subjects. Most importantly, the systemic elevation of these inflammatory markers was found to be positively correlated with the severity of AMS [58].

The enhanced feedback mechanism between oxidative stress and inflammation produces exponentially amplified damage signals. The ensuing storm of ROS and cytokines, which extensively activate endothelial cells, enhance vascular permeability, and cause tissue damage, underlie the pathology of fatal diseases such as HAPE and HACE [59].

### 3.3. Disruption of Calcium Homeostasis

Calcium ions (Ca^2+^) act as intracellular second messengers and regulate a variety of physiological processes, including muscle contraction, neurotransmission, and gene expression [60]. Physiologically, the cytosolic free Ca^2+^ is maintained at approximately 100 nM—far below the 1–2 mM extracellular Ca^2+^ concentration—through the coordination of calcium channels, calcium pumps, and exchangers, thereby establishing and sustaining a steep transmembrane electrochemical gradient [61]. However, high-altitude hypoxia disrupts this fine balance, leading to pathologically elevated cytoplasmic Ca^2+^, i.e., calcium overload. This disruption is not only a consequence of the injury, but is also a central component that drives and amplifies the cascade response to hypoxic injury [62].

The pathogenesis of hypoxia-induced calcium overload is characterized by dysfunction of the cellular calcium efflux and storage system and is exacerbated by energy depletion [63]. The Plasma Membrane Ca^2+^-ATPase (PMCA) and Sarcoplasmic/Endoplasmic Reticulum Ca^2+^-ATPase (SERCA) are critical for pumping Ca^2+^ out of the cell or into ER stores, respectively. These pumps are ATP-dependent and highly sensitive to oxidative stress. Hypoxia-induced ATP depletion and overproduction of ROS (Section 3.1) directly impair their activity, leading to impaired calcium clearance and depletion of endoplasmic reticulum calcium storage pools [64,65]. The Na^+^/Ca^2+^ exchanger (NCX) exports Ca^2+^ electrically, but hypoxic stress disrupts membrane potential and intracellular Na^+^ homeostasis [66]. Hypoxia can promote Ca^2+^ influx through multiple pathways, including activation of voltage-gated calcium channels (VGCC) and receptor-operated calcium channels (ROCC), as well as endoplasmic reticulum calcium store depletion-triggered store-operated calcium entry (SOCE) [67].

Sustained elevation of cytoplasmic Ca^2+^ can stimulate multiple destructive pathways. First, supraphysiologic levels of Ca^2+^ activate a range of calcium-dependent proteases, primarily calpains, which cleave cytoskeletal proteins, membrane receptors, and a variety of enzymes, leading to irreversible damage to cellular structures and loss of membrane integrity [65,68]. Secondly, excessive mitochondrial uptake of Ca^2+^, especially in the presence of concomitant oxidative stress, becomes a major trigger for the opening of the mitochondrial permeability transition pore (mPTP). Irreversible opening of the mPTP leads to apoptosis by collapsing the mitochondrial membrane potential, cessation of ATP production, and release of pro-apoptotic factors, such as cytochrome c. This is the key link to mitochondrial dysfunction [69,70]. Finally, Ca^2+^ overload can be mediated by the activation of Ca^2+^-dependent enzymes, such as nitric oxide synthase (NOS) and phospholipase A2, which would further stimulate the production of ROS, forming a malignant positive feedback regulation with the oxidative stress pathway described in Section 3.1 [71,72,73].

In summary, calcium overload is a key component of the hypoxia network damage response. It is activated by energy depletion and hypoxic oxidative stress, which in turn directly contributes to cellular damage and initiates the mitochondrial apoptotic program. Therefore, the development of drugs with the ability to modulate calcium homeostasis has great potential in mitigating high-altitude hypoxia injury.

### 3.4. Mitochondrial Dysfunction

Mitochondria are intracellular energy factories that provide ATP to the cell and maintain metabolic homeostasis through oxidative phosphorylation. However, in the hypoxic environment of high altitudes, they are transformed from energy providers to amplifiers of injury. Mitochondrial dysfunction is not only a part of hypoxic injury, but also a critical node where oxidative stress, inflammatory countermeasures, and calcium overload converge and amplify hypoxic injury, thereby affecting cellular function [70].

**Figure 2:**
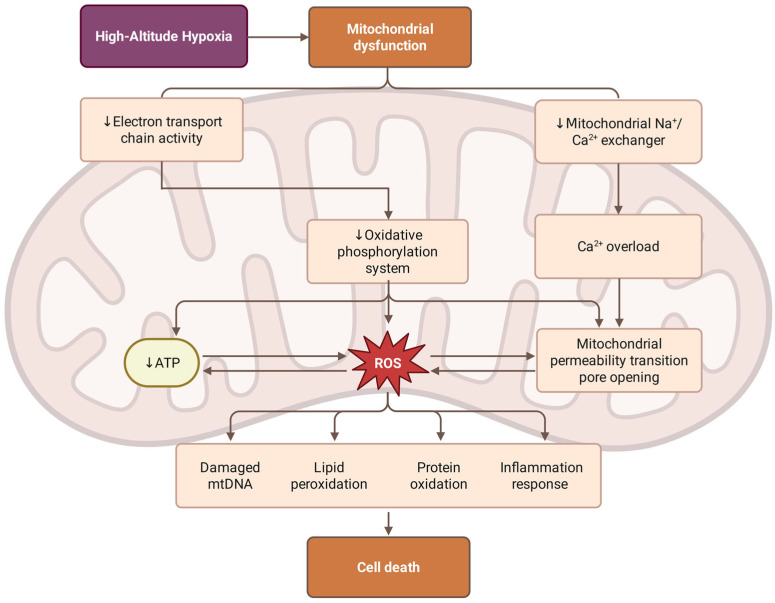
Mechanisms of hypobaric hypoxia-induced mitochondrial dysfunction. High-altitude hypoxia triggers mitochondrial dysfunction through two paths: the suppression of electron transport chain (ETC) activity, which impairs the oxidative phosphorylation system and reduces ATP production; and the inhibition of the mitochondrial Na^+^/Ca^2+^ exchanger, leading to Ca^2+^ overload and the opening of the mitochondrial permeability transition pore (mPTP). The resulting excessive accumulation of reactive oxygen species (ROS) can lead to damage, including mitochondrial DNA (mtDNA) degradation, lipid peroxidation, protein oxidation, and the activation of inflammatory responses. These bioenergetic and oxidative stressors synergistically culminate in cell death.

The primary mechanism by which high-altitude hypoxia induces mitochondrial dysfunction is disruption of the electron transport chain and oxidative phosphorylation, as illustrated in Figure 2. Under hypoxic conditions, a severe shortage of oxygen supply as the final electron acceptor for complex IV (cytochrome c oxidase) leads to an accumulation of electrons in the upstream complexes, especially complexes I and III, which results in insufficient oxygen reduction and the generation of superoxide anion [74]. The reduction in the efficiency of ATP synthesis forces the cell to rely more and more on anaerobic glycolysis, which is very inefficient and leads to metabolic acidosis and lactate buildup.

The above mechanisms of hypoxic injury create a destructive mitochondria-centered cycle. As described in Section 3.3, calcium overload directly affects mitochondrial function through multiple pathways. Mitochondrial uptake of excess calcium ions, Ca^2+^, through the mitochondrial calcium uniporter (MCU) disrupts the electrochemical gradient on both sides of the inner mitochondrial membrane [75]. In response to oxidative stress and ATP depletion, calcium influx opens the mitochondrial permeability transition pore (mPTP), which permits unrestricted passage of molecules up to 1.5 kDa across the inner mitochondrial membrane, leading to mitochondrial swelling, collapse of the membrane potential, cessation of ATP production, and, consequently, cell death [70]. Sustained opening of mPTP is an irreversible process in the apoptotic cascade reaction [76]. It leads to the release of pro-apoptotic factors (including cytochrome c, apoptosis-inducing factor (AIF) and endonuclease G) from the membrane gap into the cytoplasm. In particular, cytochrome c, which forms an apoptosome with Apaf-1 and procaspase-9, initiates the intrinsic apoptotic pathway that ultimately leads to cell death [77,78]. In addition, mitochondrial DNA (mtDNA) released as a result of mitochondrial damage acts as a damage-associated molecular pattern (DAMP) that further activates the inflammatory response via Toll-like receptor 9 (TLR9) and participates in the inflammatory cascade described in Section 3.2 [79].

The bioenergetic crisis caused by mitochondrial dysfunction not only results in immediate cell death, but also affects all energy-dependent cellular processes, including the maintenance of ionic gradients, protein synthesis and DNA repair mechanisms. This energy depletion particularly affects organs with high metabolic demands, such as the brain, heart, and kidneys, making them unusually sensitive to hypoxia. In addition, dysfunctional mitochondria become net producers of ROS and are unable to efficiently produce energy, thereby creating a damaging cycle of oxidative damage and further mitochondrial dysfunction.

### 3.5. Nitric Oxide and Erythropoietin Regulation in Hypoxia

In addition to the mechanisms described above, endogenous regulatory mechanisms such as nitric oxide (NO) and erythropoietin (EPO) play key roles in the body’s adaptive and maladaptive responses to hypoxia.

Nitric oxide plays a multifaceted role in hypoxic adaptation, functioning as a critical vasodilator, oxygen delivery enhancer, and cytoprotective molecule. NO is synthesized by three isoforms of nitric oxide synthase (NOS): endothelial NOS (eNOS), neuronal NOS (nNOS), and inducible NOS (iNOS), each contributing differently to hypoxic responses [80]. eNOS is the principal vascular isoform mediating flow- and shear-dependent vasodilation. nNOS contributes importantly to neurovascular coupling and cerebral vasodilation, and iNOS is induced in inflammatory settings and can generate much larger amounts of NO, with potential protective or deleterious effects depending on context [81]. Under hypoxic conditions, the main adaptive function of NO is to improve local blood flow and oxygen delivery through vasodilation rather than directly increasing arterial oxygen content (CaO_2_) [82]. The classic mechanism is to activate soluble guanylyl cyclase (sGC) and increase intracellular cGMP, leading to smooth muscle relaxation and increased blood flow [83]; this helps reduce pulmonary vascular resistance and limit hypoxic pulmonary vasoconstriction. This pathway is closely related to the pathogenesis of HAPE. Empirical studies on high-altitude-adapted populations have shown that Tibetans and other long-term high-altitude residents exhibit higher NO levels or NO metabolites in the lungs or circulation, which is related to their relatively lower pulmonary artery pressure and greater peripheral blood flow, suggesting that increasing NO bioavailability is an important mechanism for long-term adaptation to chronic hypoxia rather than just increasing hemoglobin [84].

Although NO contributes to hypoxic adaptation through vasodilation and cytoprotection, its role in hypoxic injury is contingent on NO concentration, hypoxia severity and cell type, among others. Under physiological concentrations, NO attenuates oxidative stress and stabilizes hypoxia-responsive signaling. However, during hypoxia-induced mitochondrial dysfunction and inflammatory activation, excess superoxide (O_2_^−^) is generated, which rapidly reacts with NO to form peroxynitrite (ONOO^−^). ONOO^−^ is a potent reactive nitrogen species capable of inducing protein tyrosine nitration, lipid peroxidation, mitochondrial enzyme inhibition, and DNA strand breaks, thereby shifting NO from a protective mediator to a contributor to cellular injury [85]. This transformation from a protective to a damaging molecule illustrates that the net physiological effect of NO depends on the balance between NO bioavailability and oxidative burden rather than on NO levels alone.

Erythropoietin is the principal hormone regulating red blood cell production and represents a cornerstone of long-term adaptation to high-altitude hypoxia [86]. The erythroid growth factor erythropoietin (EPO) is mainly produced by the kidneys in adult mammals and induces expansion of erythroid cells and iron use for hemoglobin synthesis, with its production regulated through the hypoxia-inducible factor (HIF) pathway. Under hypoxic conditions, HIF-1α and HIF-2α are stabilized and translocate to the nucleus, where they bind to hypoxia response elements in the EPO gene promoter, dramatically increasing EPO transcription. The adaptive significance of EPO-stimulated erythropoiesis lies in its ability to increase red blood cell (RBC) mass and hemoglobin concentration, thereby enhancing arterial oxygen content and compensating for reduced arterial PaO_2_ at high altitude [87]. Observations in Andean and Tibetan highlanders confirm elevated endogenous EPO levels as a marker of successful acclimatization [88,89]. However, this hematologic adaptation evolves gradually, requiring days to weeks for stem cell commitment, erythroid differentiation, and maturation to exert measurable physiological benefits [90,91]. Consequently, EPO elevation plays little role in acute altitude illnesses such as AMS or HAPE, but it is essential for chronic adaptation [92,93].

Nevertheless, excessive erythropoiesis can become maladaptive. In chronic mountain sickness (CMS), persistent EPO stimulation leads to excessive polycythemia, blood hyperviscosity, pulmonary hypertension, and right ventricular overload [94]. Recent studies indicate that this pathological overactivation may result from dysregulated HIF-2α signaling, epigenetic modifications in the VHL promoter, or imbalances in soluble EPO receptor availability, which together disrupt the normal feedback control of erythropoiesis [95]. Thus, EPO-mediated erythropoiesis exemplifies the fine balance between beneficial acclimatization and maladaptive overcompensation at high altitudes.

### 3.6. Organ Pathology and Complex Syndromes

The co-related mechanisms of hypoxia-induced damage described above are not isolated, but gather to produce complex multi-organ syndromes, which are characteristic of high-altitude diseases. The combination of oxidative stress, inflammation, calcium overload, and mitochondrial dysfunction creates organ-specific pathology that manifests itself in diverse clinical disorders, including HAPE, HACE, and CMS. This chapter will explore how these molecular and cellular mechanisms lead to patterns of organ-specific damage while maintaining systemic interconnections that exacerbate disease severity.

#### 3.6.1. Brain Pathology: High-Altitude Cerebral Edema (HACE)

The brain is a particularly vulnerable target for hypoxic injury due to its high metabolic demands, limited glycolytic capacity, and the critical role of the blood–brain barrier (BBB) in maintaining homeostasis in the brain [96,97]. HACE, the most severe neurological manifestation of high-altitude exposure, demonstrates how co-related injuries converge through multiple pathophysiological pathways to produce life-threatening brain edema.

The primary mechanism of high-altitude brain edema is the disruption of the integrity of the blood–brain barrier through oxidative stress and inflammatory responses. Hypoxia-induced generation of ROS (mainly from activated brain microglia and endothelial NOXs) directly attacks tight junction proteins (occludin, ZO-1 and claudin-5) that maintain the impermeability of the blood–brain barrier. At the same time, NF-κB–mediated inflammatory signaling, primarily in activated microglia and astrocytes, promotes the release of pro-inflammatory cytokines such as TNF-α and IL-1β. These cytokines are known to induce the expression and activation of matrix metalloproteinases, particularly MMP-9, which contributes to the degradation of tight junction proteins and the extracellular matrix surrounding cerebral capillaries. This dual attack on the structural integrity of the blood–brain barrier allows plasma proteins and water to leak into the brain parenchyma, resulting in the formation of vasogenic edema with specific symptoms of altered consciousness and neurological dysfunction [98,99,100]. Also, mitochondrial dysfunction in neurons and glial cells is a second pathway to brain edema [101]. The high demand for ATP in neural tissues makes brain cells extremely sensitive to hypoxia-induced energy failure. As a result of impaired mitochondrial oxidative phosphorylation, neurons lose the ability to maintain ionic gradients, particularly the Na^+^/K^+^-ATPase pump, and the resulting accumulation of intracellular sodium ions drives osmotic water uptake and the formation of cytotoxic edema at the cellular level [97,102]. In addition, neuronal calcium overload is exacerbated by failure of the energy-dependent calcium pump, triggering excitotoxic pathways that amplify cellular swelling and neuronal death [103]. The combination of vasogenic and cytotoxic mechanisms creates the typical case profile of high-altitude brain edema, in which symptoms that begin with headache and confusion of meaning rapidly progress to ataxia, altered mental status, and fatal brain herniation.

#### 3.6.2. Pulmonary Pathology: High-Altitude Pulmonary Edema (HAPE)

High-altitude pulmonary edema is the most serious acute lung disease in high-altitude areas and is characterized by noncardiogenic pulmonary edema that can rapidly progress to respiratory failure [104]. The pathogenesis of high-altitude pulmonary edema results from the destruction of the alveolar-capillary wall by the interaction of oxidative stress and inflammatory response.

The cause of high-altitude pulmonary edema is heterogeneous hypoxic pulmonary vasoconstriction (HPV), which results in uneven distribution of pulmonary blood flow [105]. The relatively damaged areas of vasoconstriction become hyperperfused and have elevated capillary pressures, resulting in mechanical stresses that exceed the structural limits of the alveolar-capillary membranes. These mechanical stresses, termed “stress failure,” lead to destruction of the capillary endothelium and alveolar epithelium and the formation of hyperpermeable interstitial spaces, which allow the influx of protein-rich fluid into the alveolar space [6,106,107]. Mechanical damage triggers an intense inflammatory response that amplifies the initial injury. The damaged pulmonary capillary endothelium releases DAMPs, which activate alveolar macrophages and recruit neutrophils at the site of injury [108,109]. These activated inflammatory cells produce large amounts of ROS through their NOX system, creating an “oxidative burst” that further disrupts the alveolar-capillary barrier. At the same time, NLRP3 inflammasomes in lung epithelial cells are activated to promote the release of IL-1β and IL-18, which enhances vascular permeability and maintains the inflammatory cascade response. However, it is important to note that the role of inflammasomes in the development of HAPE needs to be further clarified. Pulmonary vascular smooth muscle cells exhibit calcium overload under hypoxic conditions, leading to sustained and excessive vasoconstriction, which maintains a pressure gradient and results in capillary stress failure. In addition, calcium-dependent activation of endothelial nitric oxide synthase produces both protective nitric oxide and deleterious peroxynitrite in an oxidizing environment, leading to endothelial dysfunction [110].

#### 3.6.3. Cardiac Pathology: High-Altitude Heart Disease

The heart faces a unique set of challenges in high altitude areas, arising from both the direct effects of hypoxia and the indirect consequences of pulmonary vascular disease, which together provoke ATP depletion and oxidative stress that impair calcium-handling proteins such as SERCA and ryanodine receptors, leading to intracellular calcium accumulation [111]. Excess cytoplasmic Ca^2+^ triggers an abnormal release of calcium ions from the sarcoplasmic reticulum, producing a delayed afterdepolarization that manifests as a potentially fatal ventricular arrhythmia [112,113]. This mechanism accounts for the increased incidence of sudden cardiac death in high-altitude areas [114]. Mitochondrial dysfunction in cardiomyocytes leads to a bioenergetic crisis that directly impairs cardiac contractility. The cardiac dependence on aerobic metabolism makes it sensitive to hypoxia-induced ATP depletion, and as oxidative phosphorylation is impaired, the heart attempts to compensate by anaerobic glycolysis, but this metabolic shift produces lactate accumulation and intracellular acidosis, which further inhibits contractile function. The resulting reduction in cardiac output exacerbates systemic hypoxia and creates a vicious cycle of deteriorating cardiac function. Hypoxic pulmonary vasoconstriction leads to elevated pulmonary vascular resistance, which increases right ventricular afterload. Initially, the right ventricle compensates by hypertrophy and increased contractility. However, persistent exposure to high altitude areas can lead to progressive right heart failure because the mechanisms of myocardial injury described above impair the heart’s ability to compensate [115]. The combination of direct hypoxic injury and cardiac overload creates a lethal combination that underlies the cardiovascular deaths associated with chronic high-altitude exposure.

#### 3.6.4. Systemic Integration and Co-Related Amplification

The organ-specific pathologies described above do not occur in isolation but are interconnected through systemic networks that amplify the overall pathological burden. The development of HAPE creates an overall hypoxic crisis and accelerates damage to other organs. Impaired pulmonary gas exchange decreases arterial oxygen saturation, exacerbating cerebral hypoxia and the development of HACE. Similarly, cardiac dysfunction reduces oxygen delivery to all organs, creating a positive feedback loop that accelerates multi-organ failure. This inter-organ amplification explains why severe high-altitude disease often manifests as both HAPE and HACE, rather than single-organ injury [26]. A localized inflammatory response in a single organ leads to a systemic inflammatory state characterized by elevated circulating cytokines, complement activation, and enhanced coagulation. This generalized inflammation produces a “second strike” phenomenon, in which circulating inflammatory factors damage organs that are not experiencing direct hypoxic stress [48]. For example, severe high altitude illnesses typically result in liver and kidney dysfunction, even though these organs are relatively tolerant to hypoxia. In the most severe cases, the combination of cardiac dysfunction, increased vascular permeability, and systemic inflammation can lead to distributive shock and multiorgan dysfunction. Dysregulation of cardiovascular homeostasis leads to a cascade of organ dysfunction that rapidly becomes irreversible, which explains the high mortality rate of severe high altitude illnesses in the presence of delayed descent or treatment [99].

In summary, the organ pathology of high-altitude disease is a comprehensive manifestation of the co-related injuries described in this chapter, where the integration of oxidative stress, inflammation, calcium dysregulation, and mitochondrial dysfunction is an organ-specific pattern of injury while maintaining systemic interconnections that can rapidly progress to life-threatening multiorgan failure. These integrated pathways provide the basis for the development of comprehensive therapeutic strategies that both address the underlying mechanisms and clarify their complex interactions across organ systems.

## 4. Intervention Strategies for Damage Prevention

**Figure 3:**
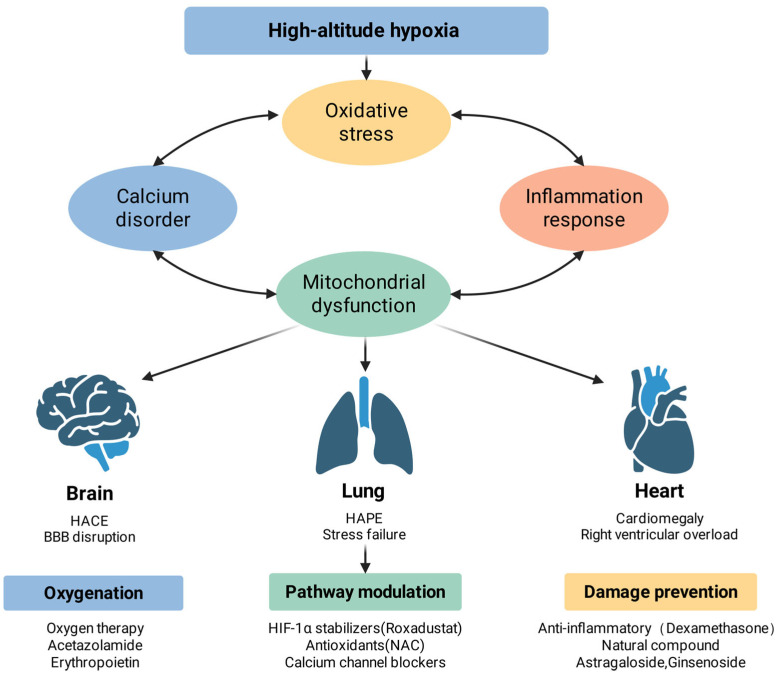
High-altitude hypoxic injury: complementary mechanisms and potential interventions. High-altitude hypoxia initiates a cascade of pathophysiological events, including oxidative stress, calcium homeostasis disorder, inflammatory response, and mitochondrial dysfunction, which interact to exacerbate cellular injury. These systemic disturbances manifest as specific organ pathologies: in the brain, they lead to high-altitude cerebral edema (HACE) and blood–brain barrier (BBB) disruption; in the lungs, they cause high-altitude pulmonary edema (HAPE) and stress failure; and in the heart, they result in cardiomegaly and right ventricular overload. Therapeutic strategies are categorized into three dimensions: oxygenation support (e.g., oxygen therapy, acetazolamide, and erythropoietin), molecular pathway modulation (e.g., HIF-1α stabilizers, antioxidants like NAC, and calcium channel blockers), and damage prevention (e.g., dexamethasone and natural compounds such as astragaloside and ginsenoside).

Understanding the co-related mechanisms of high-altitude hypoxic injury lays the foundation for the development of targeted therapeutic interventions. Effective therapeutic strategies should not only focus on isolated pathways but should also take into account the interrelationships between oxidative stress, inflammatory responses, calcium dysregulation, and mitochondrial dysfunction. This chapter systematically describes intervention strategies at three levels: upstream oxygenation and enhancement, midstream pathway regulation, and downstream damage repair mechanisms. Figure 3 can help in understanding these approaches.

### 4.1. Oxygen Supplementation Strategies

#### 4.1.1. External Oxygen Supply

Descent is currently the most effective treatment for AMS and HACE; however, descent is not necessary in all cases, and in addition to this, delivery of oxygen by nasal cannula or mask provides a suitable alternative to descent [116]. The use of low-flow oxygen (1–2 L/min) for more than 2 h rapidly increases arterial oxygen saturation and relieves the symptoms of acute altitude sickness, altitude cerebral edema, and altitude pulmonary edema based on the principle that immediate restoration of the partial pressure of oxygen reduces hypoxic stress on cellular metabolism and prevents further activation of the damage cascade network [99,117]. Hyperbaric oxygen therapy (HBOT) is the delivery of 100% oxygen at a pressure of more than one standard atmosphere, usually 2.0–3.0 ATA, thereby increasing the amount of dissolved oxygen in plasma, resulting in a plasma partial pressure of oxygen of 1500–2000 mmHg. Several studies have shown that cognitive impairment caused by altitude sickness can be effectively alleviated with hyperbaric oxygen therapy [118,119]. HBOT is now widely used for the treatment of decompression illnesses and carbon monoxide poisoning, but this equipment is technically and logistically impossible to deploy in high-altitude areas. What is actually used in high-altitude areas is portable hyperbaric chambers [116], which simulate a drop in altitude by increasing the air pressure in the chamber either manually or by electric pumps. Most products need to be used with an oxygen concentrator, and some of the products even use air compression directly to supply oxygen, without delivering 100% pure oxygen.

#### 4.1.2. Internal Oxygen Enhancement

Acetazolamide, a carbonic anhydrase inhibitor, remains the drug of choice for the prevention and treatment of acute altitude sickness [116]. In addition to its diuretic effect, it triggers metabolic acidosis by inhibiting the enzyme carbonic anhydrase, which leads to renal excretion of bicarbonate; this acidosis stimulates peripheral and central chemoreceptors, thus increasing minute ventilation and improving arterial blood gas and oxygenation [120]. In addition, the drug reduces cerebrospinal fluid secretion, thus effectively preventing HACE [121].

Nitric oxide (NO) plays a key role in the body’s adaptation to hypoxic environments, and higher levels of NO are detected in Tibetan populations [122]. In contrast, sea-level populations experience a significant decrease in NO levels after rapid entry into high-altitude environments, often accompanied by symptoms of AMS [123,124]. Dietary nitrate (NO_3_^−^), a naturally occurring active compound in a variety of foods, is particularly abundant in beet and can be converted through the body’s complex metabolic pathways to produce NO. NO is involved in several physiological processes, such as immune regulation and neurotransmission, and one of the core functions of NO is to mediate vasodilatation, thereby promoting blood circulation and oxygen transport to tissues [125], which has positive effects on the maintenance of cardiovascular homeostasis, the enhancement of athletic ability and the optimization of cognitive function. Given that the vasodilatory effect of NO improves the efficiency of oxygen delivery in hypoxia, supplementation with beet juice has been shown to be effective in reducing the impairment of cardiorespiratory endurance caused by hypoxia [126].

The administration of erythropoietin (EPO) stimulates the production of red blood cells, thereby increasing the ability to carry oxygen, but its onset of action is delayed, so it is only indicated for planned pre-acclimatization prior to high-altitude exposure, not for the treatment of acute altitude sickness [127]. Recent studies have shown that endogenous EPO levels are significantly elevated in populations living in high-altitude areas for long periods of time, confirming its important role in pre-acclimatization [128]. In addition, although hemoglobin-based oxygen carriers (HBOCs) and perfluorocarbons (PFCs) theoretically have the potential to deliver oxygen rapidly by physically dissolving or binding oxygen without relying on red blood cells, most of the developed products have not been approved in clinical trials [129,130]. Chen and Yang [131] point out in their article that although some HBOC products are still under active clinical research, most of the developed products failed to pass the phase III trial, and their effectiveness in high-altitude disease treatment needs more in-depth and systematic research.

#### 4.1.3. Mitochondrial Function Optimization

An inability of cells to utilize oxygen efficiently may produce hypoxic injury even when oxygen delivery is adequate. Mitochondrial dysfunction, characterized by disruption of the electron transport chain, excessive ROS production, and energy depletion, is both a consequence of hypoxic injury and a downstream driver of injury [132]. Therefore, protection or enhancement of mitochondrial function is an effective intervention, especially for chronic exposures, where mitochondrial adaptation determines long-term efficacy.

In hypoxia, the mitochondrial electron transport chain (ETC) becomes a major source of ROS as electrons accumulate in complexes I and III due to insufficient oxygen as the ultimate electron acceptor. Supplementation with coenzyme Q10 aims to improve the efficiency of electron transfer between complexes, reducing electron leakage and superoxide production. Coenzyme Q10 also acts as a potent lipid-soluble antioxidant for mitochondria, directly neutralizing ROS within the mitochondrial membrane to protect lipids and proteins from damage [133]. Clinical trials in a variety of diseases (heart failure, neurodegenerative diseases) have shown efficacy, but evidence for high-altitude applications is limited to small studies suggesting that supplementation with 200 mg of CoQ10 daily may reduce oxidative stress markers and improve athletic performance in high-altitude areas [134].

In addition to protecting existing mitochondria, it also promotes mitochondrial biosynthesis, which improves cellular oxidative capacity and hypoxia tolerance. Peroxisome proliferator-activated receptor gamma coactivator 1α (PGC-1α) is a master regulator of mitochondrial biogenesis that coordinates the expression of nuclear and mitochondrial genes encoding ETC components [135]. Natural compounds, including resveratrol (grape polyphenol), quercetin (flavonoids), and AICAR (activator of AMP kinase), activate PGC-1α through a variety of upstream pathways to promote mitochondrial proliferation [136,137]. However, mitochondrial biosynthesis is a slow process that takes days to weeks, which makes these interventions more suitable for pre-adaptation or chronic adaptation rather than acute treatment. In addition, evidence from human studies is limited, with most data coming from cell cultures or animal models. Notably, a more promising means may be exercise training, which naturally activates PGC-1α and is the most effective stimulus for mitochondrial biosynthesis, suggesting that physical training prior to exposure to high-altitude environments may be more effective than pharmacologic approaches [138].

### 4.2. Midstream Intervention and Modulation

Upstream oxygenation therapeutic measures are designed to target initial hypoxic injury, but the co-related mechanisms of injury described above suggest that hypoxic injury is amplified through multiple positive feedback loops involving oxidative stress, inflammation, and calcium imbalance. Thus, midstream interventions target these amplification mechanisms at key regulatory nodes, aiming to interrupt positive feedback loops before tissue damage becomes irreversible.

#### 4.2.1. HIF-1α Pathway Modulation

Hypoxia-inducible factor-1α (HIF-1α) has been experimentally verified to regulate more than 300 genes and has over 2000 suggested genes, covering angiogenesis, erythropoiesis, glycolysis, iron metabolism, inflammation and other hypoxic responses [139,140]. HIF-1α stabilizers, primarily PHD inhibitors, prevent HIF-1α degradation even under normoxic conditions, and have an opportunity for application in pre-acclimatization prior to exposure to altitude. Roxadustat is an HIF-PHI inhibitor used in the treatment of renal anemia. Prolyl hydroxylase (PHD) is the rate-limiting enzyme for the catabolism of hypoxia-inducible factor (HIF), a regulator of erythropoietin (EPO). Roxadustat improves hypoxia symptoms by decreasing the rate of HIF degradation by PHD, stabilizing the expression of HIF protein, which in turn promotes the expression of EPO and increases the erythrocyte and hemoglobin content. Guo and Li conducted a study on the preventive effect of Roxadustat on hypoxic injury during rapid ascent to high-altitude areas, which showed that Roxadustat significantly reduced hypoxia-induced inflammation, oxidative stress, and tissue damage, suggesting that it can significantly improve the body’s adaptive capacity to high-altitude exposure [141].

#### 4.2.2. Antioxidant and NOX Inhibition

N-acetylcysteine (NAC) is a precursor for glutathione synthesis and provides cysteine, the rate-limiting amino acid for glutathione production. NAC also exerts a direct antioxidant effect through its free thiol moiety and partially reverses oxidative damage by reducing disulfide bonds in oxidized proteins [142]. Studies of NAC in high-altitude environments have yielded mixed results: some studies have reported reductions in oxidative stress biomarkers (malondialdehyde, etc.), whereas others have found that its effects remain limited in clinical trials for different pathologic conditions [143]. This inconsistency likely reflects differences in dosage, timing, and patient populations. NAC has an excellent safety profile [144] and, therefore, despite its variable efficacy, remains a reasonable prophylactic option, especially for populations known to be susceptible to oxidative stress.

Tea polyphenols, particularly catechins (especially epigallocatechin gallate, EGCG) in green tea, have potent antioxidant activity through a variety of mechanisms: they directly scavenge ROS, activate the Nrf2-ARE pathway to up-regulate endogenous antioxidant enzymes (SOD, CAT and GPx), and inhibit the activation of the NLRP3 inflammatory vesicle, disrupting oxidative-inflammatory positive feedback regulation as described above. The phenolic hydroxyl structure of the EGCG molecule acts as an electron acceptor and free radical scavenger [145], thereby inhibiting the generation of reactive oxygen species (ROS) and attenuating oxidative stress-induced damage. Additionally, EGCG scavenges peroxynitrite, a highly reactive molecule that leads to the nitration of tyrosine residues in platelets, thereby reducing oxidative stress-related damage [146]. At the same time, its antioxidant effect extends to the mitochondrial level, protecting mitochondrial function [147]. Anthocyanins, abundant in blueberries, black goji berries, and purple sweet potatoes, provide potent antioxidant activity, protect vascular endothelium, improve microcirculation, and exert anti-inflammatory and anti-thrombotic effects. Black goji berry (*Lycium ruthenicum* Murray), a traditional food ingredient from the Tibetan and Qinghai regions, contains a high anthocyanin content, far exceeding that of ordinary blueberries [148]. In the Caenorhabditis elegans model, the anthocyanins in black wolfberry significantly enhanced the activities of the antioxidant enzymes superoxide dismutase and catalase, increased the glutathione (GSH)/glutathione disulfide (GSSG) ratio, and exerted excellent anti-oxidative stress activity in nematodes [149].

Inhibition of specific ROS-generating enzymes is also an effective measure and may result in better therapeutic outcomes than scavenging ROS after formation. NOX family enzymes are the main source of ROS in endothelial cells [150], inflammatory cells, and smooth muscle, which are directly activated by hypoxia independent of mitochondrial dysfunction. Apocynin and diphenyleneiodonium (DPI) are classical NOX inhibitors that reduce ROS production, improve endothelial function and reduce inflammation; however, both compounds lack isozyme selectivity and have limited human clinical data [151]. More selective NOX inhibitors Setanaxib (GKT137831) are in clinical development for the treatment of fibrotic disease [152] and have shown promise in preclinical hypoxia models, but trials at specific altitudes are lacking. XO is another targetable source of ROS, and allopurinol, an XO inhibitor used clinically in the treatment of gout, reduces ROS generation during reoxygenation and has shown some efficacy in certain cardiovascular diseases [153]. However, its correlation with persistent hypoxia is not clear.

#### 4.2.3. Calcium Homeostasis Regulation

Dysregulation of calcium ion homeostasis is a key amplification mechanism of hypoxic injury, linking energy failure and oxidative stress to mitochondrial dysfunction and cell death. Calcium ions are important signaling molecules in a myriad of physiological processes, and global calcium inhibition has serious side effects. Therefore, effective therapeutic interventions need to selectively target pathological calcium overload while preserving normal calcium signaling.

Voltage-gated calcium channels (VGCC), particularly L-type channels, mediate calcium influx during membrane depolarization, a process that is enhanced during hypoxia by energy failure-induced alterations in depolarization and channel regulation. L-type calcium channel blockers (nifedipine, amlodipine) reduce calcium influx and prevent and treat HAPE through pulmonary vasodilation. However, their therapeutic mechanisms are multifaceted: in addition to vasodilation, calcium channel blockade attenuates calcium overload in smooth muscle cells, prevents calcium-dependent inflammatory activation, and prevents calcium-mediated cell death. In one study, the calcium channel blocker nifedipine has been shown to attenuate HAPE in patients with HAPE and imaging evidence of alveolar edema, which was associated with improved arterial oxygenation, alveolar-arterial oxygen partial pressure difference, and reduced pulmonary artery pressure [154]. In contrast, in a study of 110 Indian soldiers with high-altitude pulmonary edema, nifedipine did not result in a significant improvement in therapeutic efficacy compared with placebo [155].

Ginkgo is a tree native to China that has been used in traditional medicine for a long time. Previous studies have suggested that extracts or bioactive constituents of Ginkgo biloba may help prevent hypoxic injury, mainly through their antioxidant, anti-inflammatory and anti-apoptotic effects [156]. Recent studies have shown that ginkgolide B (GB), a terpene lactone and active constituent of Ginkgo biloba, shows protective effects on hypoxic neuronal cells. Wang and Lei [157] found that GB may protect neurons from hypoxia by maintaining Ca^2+^ homeostasis through the regulation of Ca^2+^ influx and intracellular Ca^2+^ release through in vivo and in vitro experiments.

### 4.3. Anti-Inflammation Interventions and Damage Repair

Through the measures described above, oxygenation is enhanced, and the pathway of hypoxic damage amplification is partially blocked, but a certain degree of cellular damage is inevitable, especially in cases of severe or prolonged hypoxia. Therefore, this section focuses on minimizing their inflammatory damage and promoting recovery.

#### 4.3.1. NF-κB Inhibition and Cytokine Control

Dexamethasone is the main drug for the treatment of AMS and HACE in moderate-to-severe acute altitude sickness, and can also be used as an adjuvant drug for HAPE, and its efficacy has been confirmed in many experiments [92]. Dexamethasone has a very complex mechanism of action, not only through anti-inflammatory effects, but also through the glucocorticoid receptor to regulate gene transcription, membrane stabilization and signaling pathway regulation. Dexamethasone is a glucocorticoid analog that induces IκB synthesis and inhibits NF-κB nuclear translocation, preventing NF-κB from binding to its target genes, and thus preventing the transcription of pro-inflammatory cytokines (TNF-α, IL-1β, IL-6) [158]. In the treatment of high-altitude pulmonary edema, dexamethasone, as a glucocorticoid, may inhibit hypoxia-induced pulmonary endothelial dysfunction by increasing the effectiveness of nitric oxide, thus lowering pulmonary arterial pressure, and also contributes to the diffusion of oxygen by stimulating alveolar sodium and water reabsorption, which has the effect of direct activation of endothelial nitric oxide synthase and increasing the expression of eNOS mRNA [159].

Given the central role of NF-κB in hypoxia-induced inflammation, the selection of NF-κB inhibitors is also a promising therapeutic approach. Natural compounds with NF-κB inhibitory activity and other beneficial effects, curcumin (from turmeric) and resveratrol (from grapes), both inhibit NF-κB activation through a variety of mechanisms while providing antioxidant and other cytoprotective effects. Amanda M Gonzales [160] evaluated curcumin and resveratrol by treating TNF-α-stimulated adipocytes with each compound and found that they inhibited the TNF-α-activated NF-κB signaling pathway, resulting in a significant reduction in inflammatory cytokine expression. In addition, Pan and Zhang concluded through in vivo and in vitro experiments that Tetrahydrocurcumin (THC) attenuates high-altitude brain edema in mice by inhibiting NF-κB-driven inflammation and vascular endothelial growth factor/MMP-9-mediated blood–brain barrier disruption, as well as by enhancing antioxidant defenses [161]. The excellent safety profiles of curcumin and resveratrol make them reasonable therapeutic adjuncts, but there is now insufficient evidence to confirm their efficacy in high-altitude disease.

#### 4.3.2. Damage Prevention

Astragalus is used in traditional Chinese medicine and has been shown to enhance immune function and boost stress resistance. Modern pharmacological studies have found that astragalosides, astragalus polysaccharides and flavonoids are the key active ingredients in the action of astragalus. As in the heart, astragaloside protects cardiomyocytes from hypoxic injury through a variety of complementary mechanisms: protection of mitochondrial function and ATP production, maintenance of intracellular calcium homeostasis, reduction in oxidative stress, and activation of pro-multiple signaling pathways. In terms of high-altitude diseases, astragaloside has potent anti-pulmonary hypertension effects. The compound attenuates hypoxic pulmonary vasoconstriction and prevents hypoxia-induced pulmonary vascular remodeling [162], both of which are key mechanisms in the pathogenesis of high-altitude pulmonary edema and in the development of CMS. In terms of mechanism of action, astragaloside attenuated chronic intermittent hypoxia-induced myocardial injury by regulating calcium homeostasis [163]. In addition, in a chronic hypoxia model, Zhang and Lu [164] found that astragaloside may inhibit apoptosis and autophagy through the CAPN1-mediated mTOR activation pathway, thus alleviating hypoxia-induced cardiac hypertrophy. Of course, getting good results requires continuous administration for several weeks to months, so astragalus is more suitable for chronic treatment for residents of high-altitude areas or people exposed to high-altitude areas for a long time.

Ginseng and *Panax ginseng* are widely studied traditional precious herbs, in which the terpenoids ginsenosides and *Panax ginseng* saponins are the main active ingredients that play a role. A number of different ginsenosides have been isolated and categorized into protopanaxadiol-type (Rb1, Rb2, Rc, Rd) and protopanaxatriol-type (Rg1, Re, Rf), each of which possesses a unique and complementary pharmacological activity for the protection against hypoxic injury. The ginsenoside Rg1 exhibits potent neuroprotective effects through a variety of pathways that are highly relevant to hypoxic brain injury. Rg1 modulates glutamate receptor activity and reduces excitotoxicity, a major mechanism of hypoxic neuronal injury—membrane depolarization induced by energy depletion leading to glutamate over-release and calcium influx [165]. In addition, Rg1 enhances neuronal antioxidant defenses, maintains mitochondrial function, and stabilizes neuronal calcium homeostasis [166]. The ginsenoside Rd has complementary neuroprotective effects against mitochondrial dysfunction and oxidative stress. Rd maintains mitochondrial membrane potential, reduces cytochrome c release, and inhibits apoptosis through both intrinsic and extrinsic pathways [167]. The synergistic neuroprotection provided by the combination of Rg1 and Rd is superior to the use of one of the compounds alone, supporting the traditional use of whole ginseng extracts containing multiple ginsenosides.

## 5. Conclusions and Prospect

The pathophysiological process of high-altitude hypoxic injury is a typical complex systemic disease, whose central feature lies in the co-related mechanism formed between oxidative stress, inflammatory response, calcium homeostasis imbalance and mitochondrial dysfunction. This review systematizes how the components of this damage network form a vicious cycle through mutual reinforcement, ultimately leading to multi-organ pathological damage from the brain and lung to the cardiovascular system. Furthermore, we have elucidated how compensatory mechanisms, particularly nitric oxide signaling and erythropoietin regulation, contribute to physiological adaptation under hypoxic conditions. The elucidation of these mechanisms provides a theoretical basis for the development of targeted intervention strategies, leading to a comprehensive, multilevel treatment system that ranges from direct oxygen therapy to intermediate blockades and damage repair promotion. Of particular interest is that the naturally active ingredients, represented by astragaloside and ginsenoside, show unique value in this therapeutic system due to their multi-targeting regulatory properties. The multi-targeting components they contain provide a new perspective on hypoxic injury protection through their complementary pharmacological activities.

Additionally, the current study has certain limitations: First, most existing studies focus on animal models and in vitro cell experiments, while human studies are relatively underrepresented. Second, there is limited research on individual differences, and there are significant differences in the tolerance of different populations to high altitudes, especially with regard to age and developmental stage. Emerging evidence demonstrates that neonatal and adult immune systems respond fundamentally differently to hypoxic stress at the molecular level [168]. For instance, recent studies show that cord blood monocytes display decreased HIF-1α expression under anoxic conditions, contrasting sharply with the elevated HIF-1α response observed in adult peripheral blood cells [169]. This differential regulation affects multiple cellular functions, including phagocytosis, reactive oxygen species production, and vascular endothelial growth factor secretion, suggesting that neonatal immune systems employ distinct adaptive strategies compared to adults. Further research is needed on the genetic basis of such differences, the epigenetic regulatory mechanisms, and how to formulate precise prevention and treatment programs based on individual characteristics. Most importantly, many promising interventions lack clinical evidence to support them.

In order to meet these challenges, future research should make use of modern nutritional science and multi-omics technology to screen biomarkers of susceptibility to altitude sickness, establish individual risk prediction models, and formulate personalized pre-adaptation and treatment plans based on individual characteristics, to realize the transition from “universal prevention” to “precision prevention”. In terms of interventional therapy, a multi-drug combination therapy strategy and multi-target drug research and development should be explored, particularly through in-depth studies of the synergistic mechanisms of multiple active ingredients in natural compounds. With the deepening understanding of the co-related mechanism of high-altitude hypoxia injury and the application of new technologies such as precision medicine, future research should continue to deepen the basic mechanism research and at the same time pay more attention to the clinical translation and practical application, to develop safer, more effective and more convenient preventive and treatment means to protect the health and safety of people working and living in high-altitude areas.

## Figures and Tables

**Table 1:** Classification of hypoxia.

Type	Primary Defect	Arterial PaO_2_	Arterial O_2_	Causes (Examples)	References
Hypoxic	Low inspired O_2_ pressure	Decreased	Decreased	**High Altitude**, hypoventilation	[16,17]
Hypemic	Reduced O_2_-carrying capacity	Normal	Decreased	Anemia, Carbon monoxide poisoning	[18,19,20]
Stagnant	Inadequate tissue perfusion	Normal	Normal	Heart failure, Shock, Vascular occlusion	[21,22]
Histotoxic	Impaired cellular O_2_ utilization	Normal	Normal	Cyanide poisoning	[23,24]

**Table 2:** Distinguishing Acute from Chronic High-Altitude Hypoxia.

Features	Acute Hypoxia	Chronic Hypoxia
Exposure time	hours to days	months to lifelong exposure
Major diseases	Acute Mountain Sickness (AMS), High-Altitude Pulmonary Edema (HAPE), High-Altitude Cerebral Edema (HACE)	Chronic Mountain Sickness (CMS), Pulmonary Hypertension, Right Heart Failure
Underlying Mechanisms	Rapid nerve and vascular response, Cellular energy metabolism disorder, Oxidative stress and inflammation, Ion channels and membrane potential changes	Metabolic reprogramming, Tissue fibrosis and structural remodeling, Red blood cells and blood system adaptation, Angiogenesis and remodeling
Research Focus	Rapid start mechanism for injury, emergency intervention	Regulation of adaptive mechanisms, long-term health management
References	[25,26,27,28]	[4,4,29,30,31]

## Data Availability

No new data were created or analyzed in this study. Data sharing is not applicable to this article.

## Abbreviations

The following abbreviations are used in this manuscript:

AMS	Acute Mountain Sickness
HAPE	High-altitude Pulmonary Edema
HACE	High-altitude Cerebral Edema
CMS	Chronic Mountain Sickness
NLRP3	NOD-like receptor thermal protein domain-associated protein 3
ROS	Reactive oxygen species
NF-κB	Nuclear factor kappa-light-chain-enhancer of activated B cells
HBOT	Hyperbaric oxygen therapy
ETC	Electron transport chain
NAC	N-acetylcysteine
GB	Ginkgolide B
ECCG	Epigallocatechin gallate
XO	Xanthine Oxidase
DAMPs	Damage-associated molecular patterns
mPTP	Mitochondrial Permeability Transition Pore
HIF	Hypoxia-inducible factor
NOX	NADPH oxidases
TNF-α	Tumor necrosis factor-α
IL-1β	Interleukin-1β
IL-6	Interleukin-6
SERCA	Sarcoplasmic/Endoplasmic Reticulum Ca^2+^-ATPase
PGC-1α	Peroxisome proliferator-activated receptor gamma coactivator 1α
PHD	Prolyl hydroxylase
